# External to internal cranial perfusion shifts during simulated weightlessness: Results from a randomized cross-over trial

**DOI:** 10.1038/s41526-023-00267-2

**Published:** 2023-03-28

**Authors:** Alessa L. Boschert, Peter Gauger, Anja Bach, Darius Gerlach, Bernd Johannes, Jens Jordan, Zhili Li, David Elmenhorst, Andreas Bauer, Karina Marshall-Goebel, Jens Tank, Jochen Zange, Jörn Rittweger

**Affiliations:** 1 Department of Muscle and Bone Metabolism, German Aerospace Center (DLR), Institute of Aerospace Medicine, Cologne, Germany; 2 Institute of Medical Microbiology, Immunology and Hygiene, University of Cologne, Cologne, Germany; 3 Department of Cardiovascular Aerospace Medicine, German Aerospace Center (DLR), Institute of Aerospace Medicine, Cologne, Germany; 4 Institute of Aerospace Medicine, German Aerospace Center (DLR) and Chair of Aerospace Medicine, Cologne, Germany; 5 State Key Laboratory of Space Medicine Fundamentals and Application, China Astronaut Research and Training Center, Beijing, China; 6 Forschungszentrum Jülich, Institute of Neuroscience and Medicine (INM-2), Jülich, Germany; 7 KBR, Houston, TX USA; 8 Department of Pediatrics and Adolescent Medicine, University of Cologne, Cologne, Germany

**Keywords:** Physiology, Neuroscience

## Abstract

The exact pathophysiology of the spaceflight-associated neuro-ocular syndrome (SANS) has so far not been completely elucidated. In this study we assessed the effect of acute head-down tilt position on the mean flow of the intra- and extracranial vessels. Our results suggest a shift from the external to the internal system that might play an important role in the pathomechanism of SANS.

## Introduction

The human cardiovascular system evolved to cope with the challenges imposed by terrestrial gravity. In healthy persons, cardiovascular control mechanisms maintain brain perfusion with changes in posture^1^. Moreover, fluid distribution between the vascular compartment and brain tissue is balanced, thus, preventing edema formation and intracranial pressure surges. Observations in astronauts affected by the spaceflight-associated neuro-ocular syndrome (SANS) suggest that this fine balance may be perturbed when humans leave terrestrial gravity for extended durations. SANS is associated with structural changes in the brain and optic disc edema consistent with cephalad fluid overload^2,3^. The condition is a major unresolved challenge to long-duration space missions. While neck vein congestion has been reported during spaceflight, suggesting an imbalance between arterial inflow and venous drainage^4^, however, the exact mechanisms and possible relationship to SANS are not fully understood. Strict head-down tilt (HDT) bedrest reproduces cephalad fluid shifts and optic disc edema akin to SANS^5^. A previous study showed reduced internal carotid artery flow following strict HDT bedrest^6^ with differences in the time course between persons with and without SANS-like responses. Therefore, we tested the effects of acute supine and HDT bedrest on, both, internal and external carotid and jugular flow to determine the effects of gravitational loading on arterial and venous circulation.

## Methods

We measured cerebral blood flow in 11 healthy men in a cross-over designed study to test the effects of 21-hour bedrest in the −12° HDT posture versus supine posture (0°) (clinicaltrials.gov; identifier NCT 02976168). The study was approved by the ethics committee of the regional medical board (Ärztekammer Nordrhein). In accordance with the Declaration of Helsinki all subjects gave written informed consent before participation.

We chose −12° HDT because previous studies had shown that −12° HDT has stronger impact on cerebral perfusion than the more frequently used -6° HDT^18^. Prior to the 21-hour observation interval, subjects sojourned for one day and one night in the laboratory (:envihab facility, German Aerospace Center, Cologne, Germany) to allow for accommodation.

We measured blood flow in the vertebral arteries, internal and external jugular veins, and in the internal and external carotid arteries by cine phase contrast MRI (Voxel size: 0.8 × 0.6 × 4.0 mm, position: Isocenter) using a Biograph mMR 3-Tesla scanner (Siemens, Erlangen, Germany) with a 16-channel head-neck coil. We obtained measurements at baseline (supine posture), and at 1 and 21 h of exposure to bedrest in either the supine or −12° HDT posture. Then, we segmented vessels and quantified flows with the cvi42 software (www.circlecvi.com) and with custom-made Python scripts. For statistical analyses, we ran linear mixed effect models using the package nlme in R (www.r-project.org), with time and condition as fixed effects and subject ID as random effect. Mean flow rates comprised of the average of both sides for each vessel and timepoint, respectively. Additional mixed-effect models were fitted to accommodate any potential flow lateralization to the right or left vessel, as well as a side prioritization over time. Repeated within-subject identification of the external jugular veins was impeded due to the tortuous anatomy of the vessel in the investigated plane. They were, therefore, not included in the analysis.

## Results

The mean flow rate (Table 1) in the external carotid arteries was stable during supine bedrest (baseline: 239.6 ± 18.7 ml/min; 1 h: 260.1 ± 21.1 ml/min, *P* = 0.34; 21 h: 249.1 ± 23.1 ml/min, *P* = 0.68). During −12° HDT bedrest, however, external carotid arterial flow increased by ~40% (1 h: 324.3 ± 20.5 ml/min, *P* < 0.001; 21 h: 343.9 ± 23.2 ml/min, *P* < 0.001). Internal jugular venous outflow was relatively stable during supine bedrest with a decrease on the second day (baseline: 480.0 ± 81.6 ml/min; 1 h: 455.4 ± 84.0 ml/min, *P* = 0.45; 21 h: 398.9 ± 83.8 ml/min, *P* = 0.01), however, it was elevated during −12° HDT bedrest (1 h: 589.6 ± 83.4 ml/min *P* < 0.001; 21 h: 612.5 ± 84.3 ml/min, *P* < 0.001). Mean blood flow of the internal carotid arteries was decreased during both body postures in this study (baseline: 490.7 ± 16.7 ml/min; 1 h −12° HDT: 453.1 ± 17.8 ml/min, *P* < 0.001; 21 h −12° HDT: 448.8 ± 18.3 ml/min, *P* < 0.001; 1 h 0° HDT: 457.0 ± 18.0 ml/min, *P* = 0.003; 21 h 0° HDT: 441.1 ± 18.0 ml/min, *P* < 0.001). The mean vertebral arterial flow was neither affected by HDT nor supine position (baseline: 173 ± 34.4 ml/min; 1 h −12° HDT: 171.9 ± 35.0 ml/min, *P* = 0.99; 21 h −12° HDT: 167.1 ± 35.3 ml/min, *P* = 0.99; 1 h 0° HDT: 171.2 ± 35.2 ml/min, *P* = 0.99; 21 h 0° HDT: 168.2 ± 35.2 ml/min, *P* = 0.99). The linear mixed model for lateralization showed main effects of side for the internal carotid artery and the internal jugular vein (*P* = 0.016 and *P* = 0.0090, respectively), yet no significant interaction effect (*P* > 0.80). This indicates that the mechanisms involved are not lateralized. Neither main nor interactions effects could be observed for the external carotid artery.

**Table 1:** Mean flow rates of the major cervical vessels.

Mean flow [ml/min]	Baseline	Supine		HDT	
		1 h	21 h	1 h	21 h
ECA	239.6 ± 18.7	260.1 ± 21.1	249.1 ± 23.1	324.3 ± 20.5*	343.9 ± 23.2*
ICA	490.7 ± 16.7^†^	457.0 ± 18.0*	441.1 ± 18.0	453.1 ± 17.8*	453.1 ± 17.8*
IJV	480.0 ± 81.6	455.4 ± 84.0	398.9 ± 83.8	589.6 ± 83.4*	612.5 ± 84.3*
VA	173.0 ± 34.4	171.2 ± 35.2	168.2 ± 35.2	171.9 ± 35.0	167.1 ± 35.3

Table summarizing the mean flow rate of the internal jugular veins, the external and internal carotid arteries at each of the respective positions and time points.

^†^*P* < 0.05; ^*^P < 0.001 (linear mixed effect models were used for statistical analysis); *ECA* External carotid artery, *ICA* Internal carotid artery, *IJV* Internal jugular vein, *VA* Vertebral artery

## Discussion

**Fig. 1:**
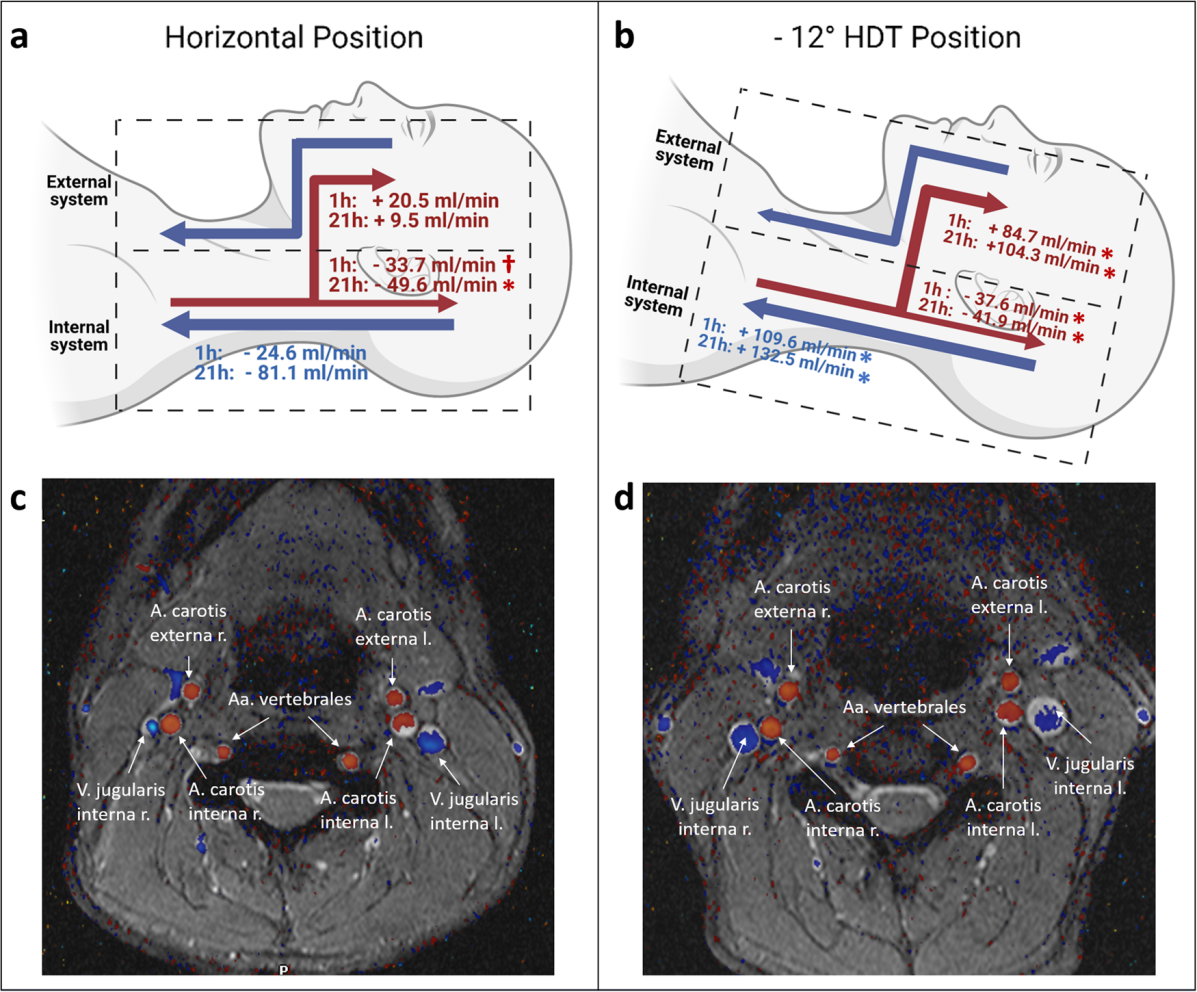
Difference in cerebral blood flow after 1 h and 21 h of the intervention. The numbers in panel **a** and **b** indicate the difference in flow in each respective position in comparison to the baseline data. The external system comprises the external carotid (red). The internal system includes the internal carotid artery (red) and jugular vein (blue). Algebraic signs refer to the physiological flow direction of each vessel. Figure panels **c** and **d** show anatomical MRI images of one of the subjects on the first interventional day in each of the respective body positions. †*P* < 0.05; *P < 0.001 (linear mixed effect models were used for statistical analysis). Panel **a** and **b** created with BioRender.com).

Overall, our study suggests that HDT bedrest, a terrestrial model for weightlessness^7^, increases external carotid flow (Fig. 1). We propose a possible explanation for venous return, the outflow maybe simultaneously increased via the external jugular vein. As previously described, the minimum cross-sectional area of the external jugular vein, the minimum and maximum cross-sectional area of the internal jugular vein were significantly increased at −12° HDT during this study^8^. One possible explanation of these findings is a shifting from the superficial carotid system towards the internal jugular veins (Fig. 1). Cerebral arterial inflow is tightly regulated by autoregulatory mechanisms, which helps to balance intracranial volume and pressure according to the Monroe-Kelly boundary conditions. Yet, autoregulation in the external carotid arteries may be less effective at offsetting negative gravitational loading during HDT^9^ and possibly spaceflight. Increased blood flow in the external carotid arteries is compensated at least partly by increased internal jugular venous outflow. However, bypassing of internal carotid autoregulation may result in additional strain on jugular outflow, which is already hampered due the HDT-induced headward hydrostatic pressure gradient. This mechanism might also result in localized intraorbital effects due to a potential collateralization between the A. angularis and A. ophtalmica as it can be observed in patients with internal carotid artery stenosis^10^. While this effect could be one of the underlying causes for SANS, flow lateralization was not observed in our study, thus not supporting the asymmetric optic disc swelling associated with SANS^3^.

While we did not observe an effect of HDT on the frontal tissue thickness^8^, the puffy face observed in astronauts^11^ and long-term bedrest studies might be a direct consequence of the identified altered fascio-cerebral perfusion: Cerebral autoregulation ensures a cerebral tissue perfusion at arteriole level under varying pressure conditions^12^. Yet, the combined effect of headward volume and tissue shifting might negatively affect the autoregulatory compensation mechanisms. Thus, a flow shift towards the external system might countervail the increased blood volume, resulting in facial edema while maintaining cerebral autoregulation and adequate cerebral perfusion.

Further physiological effects caused by HDT, such as cardio-respiratory adaptions^13,14^ intertwine with cerebral perfusion. In addition, the general HDT-induced volume shift itself might play an important role in the observed flow rates. The increased diameter of the internal jugular vein along with the altered blood flow characteristics might suggest an increase in intracranial venous pressure, which is bound to reduce the absorption of cerebrospinal fluid and flow^15,16^. Since cerebrospinal fluid bulk flow is closely linked to venous flow^17^, the increase in internal jugular blood volume might also play a role in the pathophysiology of optic disc edema development during spaceflight and strict HDT bedrest.

## Data Availability

The raw data set was generated at the German Aerospace Centre (DLR), Cologne. The anonymized data supporting the findings of this study can be requested from the corresponding author AL Boschert.
